# Dyslipidemia among adult people living with HIV on dolutegravir – based antiretroviral therapy at a private tertiary hospital in Kampala, Uganda: burden and determinants

**DOI:** 10.1186/s12879-023-08892-8

**Published:** 2024-01-05

**Authors:** Vianney John Kigongo, Joaniter I. Nankabirwa, Freddy Eric Kitutu, Ronald Ssenyonga, Ronald Kasoma Mutebi, Andrew Kazibwe, Ronald Kiguba, Andrew D. Kambugu, Barbara Castelnuovo

**Affiliations:** 1 Clinical Epidemiology Unit, School of Medicine, College of Health Sciences, Makerere University, Kampala, Uganda; 2 Infectious Diseases Institute, Makerere University, Kampala, Uganda; 3 Department of Pharmacy, Makerere University School of Health Sciences, Kampala, Uganda; 4 Department of Pharmacology and Therapeutics, Makerere University College of Health Sciences, Kampala, Uganda; 5 The AIDS Support Organisation (TASO), Kampala, Uganda; 6 Department of Medicine, Mengo Hospital, Kampala, Uganda; 7 Department of Epidemiology and Biostatistics, Makerere University School of Public Health, Kampala, Uganda; 8 Infectious Diseases Research Collaboration, Kampala, Uganda

**Keywords:** Dyslipidemia, Dolutegravir (DTG), HIV/AIDS, Total cholesterol (TC), Triglycerides (TG), Low density lipoprotein (LDL), High density lipoprotein (HDL), Uganda, Antiretroviral therapy (ART)

## Abstract

**Background:**

Understanding the burden of dyslipidemia and its associated factors among adult people living with HIV on dolutegravir (DTG) based anti-retroviral therapy (ART) is critical to provide clinical guidance and risk reduction strategies in our setting.

**Methods:**

We conducted a cross-sectional study on adult people living with HIV on DTG based ART between July and August 2022 at Mengo Hospital, a private not for profit missionary hospital owned by the Church of Uganda. Dyslipidemia was defined as: Total cholesterol (TC) ≥ 5.2 mmol/l, or high-density lipoprotein (HDL) < 1 mmol/l for men and < 1.3 mmol/l for women, or triglycerides (TG) ≥ 1.7 mmol/l, and low-density lipoprotein (LDL) ≥ 3.4 mmol/l. A participant was considered to have dyslipidemia if they had any of the lipid profile parameters in the above ranges. Socio-demographic information, clinical data and behavioral characteristics were collected. Fasting lipid profile and fasting blood glucose levels were also measured. Bivariate and multivariate analyses were done using a generalized linear model regression of the Poisson family with a log link (modified Poisson) using robust standard errors since the prevalence of dyslipidemia was more than 10%. Adjusted prevalence ratios (PR) were reported with their 95% confidence intervals (CI). A *p*-value of less than 0.05 was considered statistically significant.

**Results:**

A total of 341 participants were included. The prevalence of dyslipidemia was 78.0%, (95%CI:73.3–82.1). The highest prevalence was for low HDL (72.1%, 95%CI 67.1–76.7) followed by high TG (20.2%, 95%CI: 16.3–24.9), high TC (12.0%, 95%CI: 9.0–15.9) and high LDL (6.5%, 95%CI: 4.3–9.6). Female sex (aPR:1.55, 95%CI: 1.32–1.84, *p* < 0.001) and previous use of protease inhibitor (PI) based ART regimen (aPR:1.26, 95%CI: 1.04–1.53, *p* = 0.018) were significantly associated with dyslipidemia.

**Conclusion:**

We demonstrate that the prevalence of dyslipidemia is very high as it was present in more than three quarters of the study participants. Female sex and previous use of PI based ART regimen were significantly associated with dyslipidemia. Management of dyslipidemia should be integrated in the HIV treatment package and we recommend further inquiry into the temporal relationship between dyslipidemia and DTG among ART patients, if any.

**Supplementary Information:**

The online version contains supplementary material available at 10.1186/s12879-023-08892-8.

## Introduction

In 2021, 38 million people were estimated to be living with HIV world-wide, of whom 680,000 died from AIDS-related illnesses [1]. Sub-Saharan Africa has more than two-thirds of all people living with HIV (PLHIV) globally making it the hardest affected region in the world [2].With this region, Eastern and Southern Africa regions are the most heavily affected by HIV, accounting for approximately 55% of all PLHIV [3]. The burden of HIV in Uganda is still high with about 1.3 million adults (≥ 15 years) living with HIV by 2020, accounting for more than 90% of the national burden by age [4]. In addition, HIV related deaths were the third commonest in Uganda in 2019 [5]. However, there has been a steady increase in PLHIV on ART which has saved and prolonged many lives.

The World Health Organization (WHO) recommends the use of dolutegravir (DTG) as the preferred first-line and second-line treatment option for all PLHIV, including pregnant women and those of childbearing age. This is based on new evidence that DTG more effective, easier to take and has fewer side effects than available alternative drugs used for treatment of HIV among PLHIV [6].

Since 2018, the Uganda Ministry of Health (MoH) recommends the use of DTG in combination with tenofovir (TDF) and lamivudine (3TC) as the preferred first line regimen for treatment of adults living with HIV [7].

PLHIV on ART are known to experience several side effects and adverse drug reactions including dyslipidaemia [8]. For instance, patients on stavudine, didanosine, zidovudine, lamivudine and efavirenz, protease inhibitors (including atazanavir and ritonavir) reported elevated triglycerides (TG), total cholesterol (TC) and reduced high-density lipoprotein (HDL) [9–11]. PIs were also reported to elevate low density lipoprotein (LDL) [12, 13]. However, DTG, together with other integrase strand transfer inhibitors (INSTIs), seem to have a higher incidence of dyslipidemia compared to those on non-nucleoside reverse transcriptase inhibitors (NNRTIs) like efavirenz [14].

Additionally, clinical trials in other settings have reported a significantly greater increase in baseline total cholesterol TC and LDL among PLHIV on DTG in black African populations [15, 16].

In Uganda, studies have focused on hyperglycemia and diabetes mellitus (DM) among PLHIV on a DTG-based ART regimen [17, 18] and yet, poorly managed dyslipidemia is a risk factor for non-communicable diseases [like hypertension (HTN), diabetes (DM)] and cardiovascular disease [19].

Moreover, lipid profile levels are not routinely measured among PLHIV in Uganda due to cost. There is a degree of uncertainty on the prevalence of dyslipidemia in PLHIV on DTG-based regimens and its associated factors. The current study determined the burden and factors associated with dyslipidemia among adults receiving DTG-based ART regimens at Mengo Hospital in Kampala, Uganda.

## Methods

This was a cross-sectional study with both descriptive and analytical components carried out in the counseling and home care department of a private not for profit missionary tertiary hospital called Mengo Hospital in Kampala, Uganda from July to August 2022. The clinic operates daily and has an estimated outpatient attendance of about 400. The clinic serves about 8800 active PLHIV ART, with approximately 7000 adults on DTG-based ART. Using the systematic sampling procedure, every 4th participant was sampled and 341 adults (≥ 18-years) with confirmed HIV-positive infection were enrolled into the study upon providing written informed consent. These adults were on a DTG based ART regimen for more than 6 months and had self-reported fasting for at least 6 h.

Participants who were bed ridden, had a history of use of lipid - lowering therapy, with cardiovascular risk factors, or using lipid - affecting agents (such as thiazides, beta blockers, antipsychotics, steroids) before switch or initiation to a DTG-based ART, those who were unable to comprehend either English or Luganda (the local language used in central Uganda) were excluded from the study.

### Data collection

Two trained enumerators (diploma nurses) administered a pretested questionnaire to collect sociodemographic (age, sex, marital status, residence, education level, employment status and distance from the health facility) and behavioral characteristics i.e., smoking status, alcohol intake and physical activity. Clinical data collected were past medical history, height, weight, blood pressure (BP), fasting blood sugar (FBS), baseline CD4 + T cell count, HIV duration in years, duration on DTG-based ART in months and duration on ART in years.

Participant weight, exclusive of heavy outer garments, hair ornaments and shoes, if any, was measured using the seca weighing scale in complete kilograms. Height was abstracted from the patient files. The body mass index (BMI) was then computed as weight (in kg)/height (in m^2^).

Blood pressure (BP) was measured by a pretested automatic sphygmomanometer of mercury type in a sitting position 10 min after rest. Two measurements, 5 min apart were recorded for consistency. A third measurement was taken as final in case of a 10mmHg difference in systolic BP in the first two readings.

About 4 ml of a blood sample were aseptically collected from each participant by a qualified laboratory staff and placed in a yellow top vacutainer with clot activator for the determination of fasting lipid profile levels. Patient files were sorted basing on whether they were on DTG based ART regimen or not. In addition to being reminded about their appointment date a day before the scheduled visit, patients were also requested not to eat anything after their last meal of that day. Fasting blood sugar levels were determined using a Freestyle glucometer (Abott Laboratories, Canada) for all eligible participants. A sterile single use lancet was used to prick a participant’s disinfected finger and a small drop of the blood placed onto the glucometer strip already mounted into the glucometer. The blood sugar levels were read off and recorded in mmol/L. The results were recorded in a laboratory results tool. Venous blood samples were collected from the eligible participants early in the morning before they ate anything and were allowed to clot for at least 3 min then later centrifuged at 2400 revolutions/min for 5 min at Mengo Hospital ART clinic laboratory. The serum obtained was pipetted into cryogenic vials, stored in a refrigerator at < 4^0^ C. These were later transported under cold chain in a sample carrier with ice-packs and a temperature monitoring device by the principal investigator for analysis of lipid profile using the Cobas 3000 chemistry analyzer (Roche Diagnostics, USA) at Jinja Regional Referral Hospital, Uganda laboratory in two lots. This was done to reduce the cost of lipid analysis that was so high among laboratories in Kampala.

### Statistical analysis

Data was analysed using STATA V.14.0 (StataCorp. 2013. *Stata Statistical Software: Release 14*) in which all continuous variables were summarised as medians and ranges while the categorical variables were summarised as percentages and proportions. The prevalence of dyslipidaemia was calculated as the percentage of participants with dyslipidaemia over the total number of study participants. Data was assessed for collinearity. At bivariate and multivariate analysis, we used a generalized linear model regression of the Poisson family with a log link (modified Poisson) with robust standard errors to analyse the factors associated with dyslipidaemia among study participants since the prevalence was more than 10% [20]. In the multivariate analysis, we included variables with *p*-value of ≤ 0.2 at bivariate analysis as well as those known from literature such as age, duration on ART, physical activity, HIV duration and alcohol use. Interaction was assessed by forming two-way product terms with variables which were significantly associated with dyslipidaemias (*p* < 0.05) using the chunk test. Confounding was assessed by checking if the variables changed the estimates by greater than or equal to 10%. CIs were presented at 95% level of significance along with the *p* values. Statistical significance was considered at a *p* value of less than 0.05.

## Results

Majority of the participants were female 66.9% (228/341) with median age of 43 years (IQR: 36–50), residing in rural areas 71.3% (243/341) and married 54.8% (187/341). Of the participants enrolled, 29.9% (102/341) were obese, with median HIV duration of 9.5 years (IQR: 4.9–12.7) and median DTG duration of 35.4 months (IQR: 23.8–40.6). The majority of participants were on TDF/3TC/DTG 96.5% (329/341) and were previously on an NNRTI-based regimen 82.7% (282/341). Only 5.3% (18/341) of the participants had a high FBG and 19.4% (66/341) of them had HTN (Table 1).

**Table 1:** Sociodemographic and clinical characteristics of study participants on DTG-based ART in Mengo Hospital, Kampala, Uganda, 2022 (n = 341)

Variable		Median (Q1, Q3)	All n (%)
Sex	Female		228 (66.9)
Age		43 (36, 50)	
Marital status	Married		187 (54.8)
	Unmarried		154 (45.2)
Education level	None		21 (6.2)
	Primary		112 (32.8)
	Secondary		149 (43.7)
	Tertiary		59 (17.3)
Employment status	Unemployed		35 (10.3)
	Formally employed		54 (15.8)
	Self employed		224 (65.7)
	Informally employed		28 (8.2)
Residence	Rural		243 (71.3)
	Urban		98 (28.7)
Smoking history	Yes		3 (0.9)
Alcohol use	Yes		72 (21.1)
Physical activity	Low		209 (61.3)
BMI	Normal		145 (42.5)
	Overweight		94 (27.6)
	Obese		102 (29.9)
HTN (by BP)	Yes		66 (19.4)
FBG (≥ 7mmol/L)	High		18 (5.3)
Baseline CD4* (n = 340)		296 (149, 468)	
DTG duration (months)		35.4 (23.8, 40.6)	
HIV duration (years)		9.5 (4.9, 12.7)	
Previous ART regimen			
	NNRTI based		282 (82.7)
	PI based		16 (4.7)
	None		43 (12.6)
Current ART regimen			
	ABC/3TC/DTG		7 (2.1)
	AZT/3TC/DTG		5 (1.5)
	TDF/3TC/DTG		329 (96.5)
Familial DM	No		259 (76.0)
Familial HTN	No		227 (66.6)

* Missing data, n - number of participants, Q1–1st quartile, Q3–3rd quartile, BMI – body mass index, 3TC - lamivudine, TDF - tenofovir, AZT - zidovudine, ABC - abacavir, DTG - dolutegravir, NNRTI – non-nucleoside reverse transcriptase inhibitor, PI – protease inhibitor, HTN- hypertension, WHO - World Health Organization, ART - antiretroviral therapy, FBG - fasting blood glucose, DM - diabetes mellitus, BP-blood pressure measurement

The overall prevalence of dyslipidemia was 78.0% (95%CI 73.3–82.1). The highest prevalence was for low HDL 72.1% (95%CI 67.1–76.7) followed by high TG 20.2% (95%CI 16.3–24.9), high TC 12.0%, (95%CI 9.0–15.9) and high LDL 6.5%, (95%CI 4.3–9.6) (Table 2).

**Table 2:** Dyslipidemia among study participants on DTG-based ART in Mengo Hospital, Kampala, Uganda, 2022 (n = 341)

Variable		Mean (SD)	n (%)	95% CI
Lipid profile parameter				
	TC > = 5.2 mmol/l	3.94 (1.05)	41 (12.0)	9.0–15.9
	HDL*	1.00 (0.37)	246 (72.1)	67.1–76.7
	LDL > = 3.4 mmol/l	2.19 (1.00)	22 (6.5)	4.3–9.6
	TG > 1.7 mmol/l	1.30 (0.62)	69 (20.2)	16.3–24.9
	Overall		266 (78.0)	73.3–82.1

* <1.0 mmol/l in males and < 1.3 mmol/l in females, CI - confidence interval, n - number of participants, HDL - high density lipoprotein, TC – total cholesterol, TG – triglycerides, LDL – low density lipoprotein, SD – standard deviation

At bivariate analysis, being female (cPR:1.53, 95%CI 1.30–1.81, *p* < 0.001), having up to primary education or less (cPR – 1.14, 95%CI 1.02–1.27, *p* = 0.021), being on DTG based ART regimen for < 2 years (cPR: 1.13, 95%CI 1.01–1.26, *p* = 0.034), overweight (cPR: 1.13, 95%CI 0.98–1.31, *p* = 0.093), obese (cPR: 1.24, 95%CI 1.09–1.31, *p* = 0.001) and previous use of PI based ART regimen (cPR: 1.14, 95%CI 0.93–1.38, *p* = 0.199) had a *p*-value less than 0.2 hence they were considered for multivariate analysis (Table 3). Additionally, age, duration on ART, physical activity, duration of HIV infection, and alcohol use were forced into multivariate analysis.

**Table 3:** Bivariate analysis for sociodemographic and clinical characteristics of study participants on DTG-based ART in Mengo Hospital, Kampala, Uganda, 2022 (n = 341)

Variable		Dyslipidemia		cPR	95% CI	*P* value
		No	Yes			
Sex						
	Male	48 (42.5)	65 (57.5)	Reference		
	Female	27 (11.8)	201 (88.2)	1.53	1.30–1.81	**< 0.001**
Age	25–49	51 (21.2)	190 (78.8)	Reference		
	18–24	1 (10.0)	9 (90.0)	1.14	0.92–1.42	0.232
	≥ 50	23 (25.6)	67 (74.4)	0.94	0.82–1.08	0.415
Education level	Post primary	54 (26.0)	154 (74.0)	Reference		
	Primary or less	21 (15.8)	112 (84.2)	1.14	1.02–1.27	**0.021**
Physical activity	High	38 (23.3)	155 (76.7)	Reference		
	Low	37 (20.8)	141 (79.2)	0.56	0.92–1.16	0.306
Alcohol Use	No	55 (20.5)	`214 (79.5)	Reference		
	Yes	20 (27.8)	52 (72.2)	0.91	0.78–1.06	0.224
DTG duration (years)	≥ 2	62 (24.5)	191 (75.5)	Reference		
	< 2	13 (14.8)	75 (85.2)	1.13	1.01–1.26	**0.034**
ART duration (years)	< 10	49 (24.1)	154 (75.9)	Reference		
	≥ 10	26 (28.8)	112 (81.2)	1.07	0.96–1.20	0.237
HIV duration (years)	< 10	43 (24.2)	135 (75.8)	Reference		
	≥ 10	32 (19.6)	131 (80.4)	1.06	0.95–1.19	0.313
Baseline CD4* (n = 340)	< 200	30 (24.8)	91 (75.2)	Reference		
	200–499 ≥ 500	30 (20.6) 15 (20.3)	116 (79.4) 59 (79.7)	1.08 0.90	0.92–1.25 072–1.12	0.347 0.311
Previous ART regimen	NNRTI based	65 (23.1)	217 (76.9)	Reference		
	PI based	2 (12.5)	14 (87.5)	1.14	0.93–1.38	**0.199**
	None	8 (18.6)	35 (81.4)	1.06	0.90–1.24	0.483
BMI	normal	43 (29.7)	102 (70.3)	Reference		
	overweight	19 (20.2)	75 (79.8)	1.13	0.98–1.31	**0.093**
	obese	13 (12.8)	89 (87.2)	1.24	1.09–1.41	**0.001**
FBG	Normal	72 (22.3)	251 (77.7)	Reference		
	High	3 (16.7)	15 (83.3)	1.07	0.86–1.33	0.524

* Missing data, n- number of participants, CI - confidence interval, cPR -crude prevalence ratio, BMI – body mass index, DTG - dolutegravir, NNRTI – non-nucleoside reverse transcriptase inhibitor, PI – protease inhibitor, ART - antiretroviral therapy, FBG - fasting blood glucose

At multivariate analysis, being female (aPR:1.55, 95%CI 1.32–1.847, *p* < 0.001) and previous use of PI based ART regimen (aPR:1.26, 95%CI 1.04–1.53, *p* = 0.018) were significantly associated with dyslipidemia (Table 4).

**Table 4:** Multivariate analysis of factors associated with dyslipidemia among study participants on DTG-based ART in Mengo Hospital, Kampala, Uganda, 2022 (n = 341)

Variable		aPR	95% CI	*P* value
Sex	Male	Reference		
	Female	1.55	1.32–1.84	**< 0.001**
Previous ART regimen				
	NNRTI based	Reference		
	PI based	1.26	1.04–1.53	**0.018**
	None	1.08	0.94–1.25	1.24

CI - confidence interval, aPR – adjusted prevalence ratio, ART – antiretroviral therapy, NNRTI – non-nucleoside reverse transcriptase inhibitor, PI – protease inhibitor

## Discussion of results

The overall prevalence of dyslipidemia in the current study is 78.0% with the prevalence of low HDL cholesterol at 72.1%. This prevalence is high as three quarters of the respondents have dyslipidemia. In contrast, high TC, high LDL and high TG are relatively low, with less than a quarter of the population having any of these forms of dyslipidemia. This high prevalence of dyslipidemia (low HDL cholesterol) could reflect the high prevalence in the general population. Female participants were twice the male participants (66.9% vs. 33.1). In addition, there is a positive association between increasing age and a rise in the prevalence of low HDL, with a steeper increase among women than for men [21]. In our study, the median age was 43 years (IQR: 36–50) with more than half of the participants aged 40 years and above. Therefore, this high prevalence of dyslipidemia could have been as a result of enrolling older patients and mostly female.

Previous studies have demonstrated that initiation of NNRTI based ART regimens results in increases in HDL of approximately 40% depending on the agent used, with increases in TC, LDL and TG also seen, although the TG increases are usually not as severe as those seen with some PIs [22]. However, this finding is contrary to our results in which the prevalence of low HDL cholesterol is high among the study participants probably because not all patients respond similarly to antiretroviral regimens [23].

The high prevalence of dyslipidemia means that most of the study participants are at a risk of atherosclerotic cardiovascular disease events and could benefit from statin initiation if clinically indicated [24].

A cross-sectional study of 597 PLHIV on the prevalence of low HDL among adults receiving ART for at least 6 months in Zambia from April to December 2019 reported a high proportion of young adults (75%) and adults (58%) with low HDL who were on DTG-based ART regimens [25]. Similar studies in SSA on the prevalence of dyslipidemia among PLHIV on ART regimens other than DTG-based ones have reported prevalence ranging from 15.9 to 83.3% [26–30].

A recent cross-sectional study in Halibet National Referral Hospital and Orotta National Medical Surgical Referral Hospital Eritrea from March to June, 2018 reported a prevalence of 86.6% among PLHIV [31].

The factors that were significantly associated with dyslipidemia among study participants on DTG based ART regimen were being female and previous use of PI based ART regimen. Surprisingly, age, duration on ART, physical activity, duration of HIV infection, alcohol use, education level, being on DTG based ART regimen for less than 2 years, being overweight and being obese were not significantly associated with dyslipidemia.

Females were 1.55 times as likely to have dyslipidemia than males. This is line with findings from a cross sectional study by Benson and colleagues in 2021. Benson et al.’s study reported that females were 2.54 times more likely to have low HDL compared with males. Gender differences have been partly explained by the dramatic lipid profile changes in women than in men due to complex hormonal modifications throughout their life time, especially those related to pregnancy and menopause [32]. In addition, estrogen and testosterone have been reported to have influence on the activities of hepatic lipase which plays a role in HDL metabolism and its levels are inversely related with those of HDL [33]. These hormones respectively tend to decrease and increase hepatic lipase levels and as a result, women tend to have higher HDL levels than men [34–36].

Study participants taking a PI based ART regimen before switching to a DTG-based regimen were 1.26 times as likely to have dyslipidemia than those on a previous NNRTI based ART regimen. Integrase strand transfer inhibitors (INSTI) have been reported to have a lower incidence of dyslipidemia than ritonavir boosted protease inhibitors but higher rate compared with those on NNRTI [14]. Therefore, dyslipidemia could have existed among these participants due to previous exposure to PIs. In a large observational cohort study to determine the improvement of lipid profile after switching from efavirenz or ritonavir-boosted PIs to rilpivirine or once-daily integrase inhibitors, the TC and TC/HDL ratio significantly decreased among participants switched from PI based regimens to DTG-based regimens within one year. However, reductions of LDL and TG levels were not statistically significant [37]. In this study, baseline lipid profile before switch were not established and therefore, we could not ascertain if the dyslipidemia was due to the previous ART exposure or other factors.

### Strengths of the study

This is one of the first studies assessing dyslipidemia among patients on a DTG based regimen in Uganda thus laying a foundation for future research. Additionally, we managed to collect complete data from all variables considered in the study and only had one missing entry on CD4 count and the data collection process was standardized which increases the replicability and comparability of our findings with future studies.

### Limitations of the study

We acknowledge the following limitations. First, a questionnaire was used to collect data with self-reporting on some variables (like true fasting status, substance abuse, alcohol use, smoking and familial diseases) which increases the chances of patients reporting desirable answers. Second, there was a possibility of selection bias due to elimination of potential study participants that had taken breakfast. Third, some variables like WHO stage, viral load and diet were not studied yet they have been consistently reported to be associated with dyslipidemia in other studies. Fourth, this was a cross-sectional study and therefore a temporal relationship between dyslipidemia and the covariates could not be determined. Fifth, we excluded participants using lipid-lowering therapy, with cardiovascular risk factors, or using lipid-affecting agents (such as thiazides, beta blockers, antipsychotics, steroids); no data available from these exclusions and sixth, we were unable to determine the cumulative exposure to PIs and NNRTIs at baseline and therefore the difference in dyslipidemia risk between the two regimens couldn’t be determined.

## Conclusion

We demonstrate that the prevalence of dyslipidemia is very high as it was present in three quarters of the study participants on DTG based ART. This suggests that many people are at an increased risk of developing complications like cardiovascular diseases which arise from dyslipidemia. Female sex and previous use of PIs were significantly associated with dyslipidemia among adult patients on a DTG-based ART. We recommend further research to understand whether there is a temporal relationship between dyslipidemia and DTG among ART patients.

### Electronic Supplementary Material

Below is the link to the electronic supplementary material.

Supplementary Material 1

## Data Availability

All available data can be obtained by contacting the corresponding author.

## List of Abbreviations

3TC: Lamivudine
ABC: Abacavir
ART: Anti-retroviral therapy
AZT: Zidovudine
BMI: Body mass index
CAD: Coronary Artery Disease
CD4: Cluster of differentiation 4
CI: Confidence interval
DM: Diabetes Mellitus
DTG: Dolutegravir
HAART: Highly active antiretroviral therapy
HDL: High density lipoprotein
HIV: Human immunodeficiency virus
HTN: Hypertension
IHME: Institute for Health Metrics and Evaluation
INSTI: Integrase strand transfer inhibitor
LDL: Low density lipoprotein
MoH: Ministry of Health
NCEP: National Cholesterol Education Program
NRTIs: Nucleoside Reverse Transcriptase Inhibitors
PLHIV: People living with HIV
PR: Prevalence ratio
TC: Total cholesterol
TDF: Tenofovir
TG: Triglycerides
UNAIDS: The Joint United Nations Programme on AIDS
WHO: World Health Organization

## Declarations

### Provenance and peer review

Not commissioned; not peer reviewed.
