# Supplementary material for: Dyslipidemia among adult people living with HIV on dolutegravir – based antiretroviral therapy at a private tertiary hospital in Kampala, Uganda: burden and determinants
Source: BMC Infect Dis. 2024 Jan 5;24:53. doi: 10.1186/s12879-023-08892-8 (PMC10770888; doi:10.1186/s12879-023-08892-8)
Supplement: Supplementary file 1 — Supplementary Material 1 [file 12879_2023_8892_MOESM1_ESM.docx]

# Supplementary material

# Operational definitions

**Dyslipidemia:** dyslipidemia was defined as: TC ≥5.2 mmol/l, or HDL<1 mmol/l for men and <1.3 mmol/l for women, or TG ≥ 1.7 mmol/l, and LDL≥3.4 mmol/l (38). A participant was considered to have dyslipidemia if they had any of the lipid profile parameters in the above ranges.

**Cardiovascular diseases (CVDs):** CVDs referred to a group of disorders of the heart and blood vessels like heart attacks, coronary heart disease, strokes, venous thromboembolism, myocardial infarction and peripheral artery disease (39).

**Body mass index (BMI)**: BMI was calculated as weight (kg) divided by height (m^2^) and was used as indicator of normal weight (BMI ≤ 25.0 kg/m^2^), overweight (BMI: 25.0 – 29.9 kg/m^2^) and obesity (BMI ≥ 30.0 kg/m^2^) (40).

**Physical activity:** high physical activity was defined as achieving at least 600 minutes of physical activity per week or at least 5 days a week of any combination of any vigorous or moderate-intensity sports, fitness or leisure activities that cause a large or small increase in breathing or heart rate such as cycling, running, football, household chores, harvesting, fishing or hunting, swimming and volleyball using the WHO STEPS Instrument (41).

**Drug abuse / substance use** referred to the use of any substances other than alcohol or tobacco products such as cannabis, marijuana, cocaine, acetaminophen, khat and petrol.

**Alcohol use** referred to drinking alcohol at least three times a week as previously defined in other studies on alcohol use in Uganda (42){Kabwama, 2016 #1166}. Binge drinking referred to taking alcohol three or more times per week while moderate consumption referred to less than three occasions of alcohol per week.

**Employment status** was defined as unemployment if people were jobless, whether they were searching for a job or not; formal employment was when an individual was working in an institution or company or organization as hired employment with a binding institution contract; informal employment referred to work without a defined income (stable income) and an established working agreement; and self-employment was when a person did not work for an employer but found work for themselves, or had their own business.
